# Temporal and spatial patterns of serologic responses to *Plasmodium falciparum* antigens in a region of declining malaria transmission in southern Zambia

**DOI:** 10.1186/1475-2875-11-438

**Published:** 2012-12-31

**Authors:** Tamaki Kobayashi, Sandra Chishimba, Timothy Shields, Harry Hamapumbu, Sungano Mharakurwa, Philip E Thuma, Gregory Glass, William J Moss

**Affiliations:** 1 Department of Epidemiology, Johns Hopkins Bloomberg School of Public Health, Baltimore, MD, USA; 2 Malaria Institute at Macha, Macha Research Trust, Choma, Zambia; 3 Department of Molecular Microbiology and Immunology, Johns Hopkins Bloomberg School of Public Health, Baltimore, MD, USA

## Abstract

**Background:**

Critical to sustaining progress in malaria control is comprehensive surveillance to identify outbreaks and prevent resurgence. Serologic responses to *Plasmodium falciparum* antigens can serve as a marker of recent transmission and serosurveillance may be feasible on a large scale.

**Methods:**

Satellite images were used to construct a sampling frame for the random selection of households enrolled in prospective longitudinal and cross-sectional surveys in two study areas in Southern Province, Zambia, one in 2007 and the other in 2008 and 2009. Blood was collected and stored as dried spots from participating household members. A malaria rapid diagnostic test (RDT) was used to diagnose malaria. An enzyme immunoassay (EIA) was used to detect IgG antibodies to asexual stage *P. falciparum* whole parasite lysate using serum eluted from dried blood spots. The expected mean annual increase in optical density (OD) value for individuals with a documented prior history of recent malaria was determined using mixed models. SatScan was used to determine the spatial clustering of households with individuals with serological evidence of recent malaria, and these households were plotted on a malaria risk map.

**Results:**

RDT positivity differed markedly between the study areas and years: 28% of participants for whom serologic data were available were RDT positive in the 2007 study area, compared to 8.1% and 1.4% in the 2008 and 2009 study area, respectively. Baseline antibody levels were measured in 234 participants between April and July 2007, 435 participants between February and December 2008, and 855 participants between January and December 2009. As expected, the proportion of seropositive individuals increased with age in each year. In a subset of participants followed longitudinally, RDT positivity at the prior visit was positively correlated with an increase in EIA OD values after adjusting for age in 2007 (0.261, p = 0.003) and in 2008 (0.116, p = 0.03). RDT positivity at the concurrent visit also was associated with an increase in EIA OD value in 2007 (mean increase 0.177, p = 0.002) but not in 2008 (−0.063, p =0.50). Households comprised of individuals with serologic evidence of recent malaria overlapped areas of high malaria risk for serologic data from 2009, when parasite prevalence was lowest.

**Conclusions:**

Serological surveys to whole asexual *P. falciparum* antigens using blood collected as dried blood spots can be used to detect temporal and spatial patterns of malaria transmission in a region of declining malaria burden, and have the potential to identify focal areas of recent transmission.

## Background

Increased funding for malaria control and elimination has led to implementation of comprehensive control programmes and concomitant reductions in the burden of malaria in many parts of sub-Saharan Africa [1,2]. Zambia has been a model country for malaria control within sub-Saharan Africa and has achieved a significant decline in the burden of malaria [3,4]. Zambia’s national malaria control programme includes provision of artemisinin-based combination therapy, distribution of insecticide-treated nets, indoor residual spraying in urban and peri-urban areas, and intermittent preventive treatment of pregnant women [3,5]. By 2008, the prevalence of parasitaemia and severe anaemia in children between six and 59 months of age decreased by 53% and 68%, respectively, compared with levels in 2006 [3]. In April 2009, the World Health Organization announced that Zambia reached the 2010 Roll Back Malaria target of greater than 50% reduction in malaria mortality compared to levels in 2000 [6].

Despite this impressive achievement, the incidence of malaria increased in five of nine provinces of Zambia in 2010 [4,7]. The greatest resurgence occurred in Eastern and Luapula Provinces, where the number of reported cases of malaria doubled from levels in 2008 [4]. Such trends highlight the challenge of sustaining effective malaria control. Critical to such control is effective surveillance to identify outbreaks, target control efforts and prevent resurgence. Serologic responses to *Plasmodium falciparum* can serve as a proxy measure of malaria transmission [8-13] and may be a useful tool for enhanced surveillance in the pre-elimination phase of malaria control. Measurement of antibodies to single parasite antigens such as MSP-1_19_ and AMA-1 identified infection within the previous four months among children younger than six years of age in The Gambia [14]. Serologic surveillance may be feasible on a large scale using blood collected on filter paper [15] or oral fluid samples [16,17]. IgG antibody levels to whole, asexual *P. falciparum* lysate were measured by enzyme immunoassay in two community-based cohorts in southern Zambia to assess the utility of serological surveys to identify temporal and spatial patterns of recent malaria transmission in a region with declining malaria burden in southern Zambia.

## Methods

### Study site

**Figure 1:**
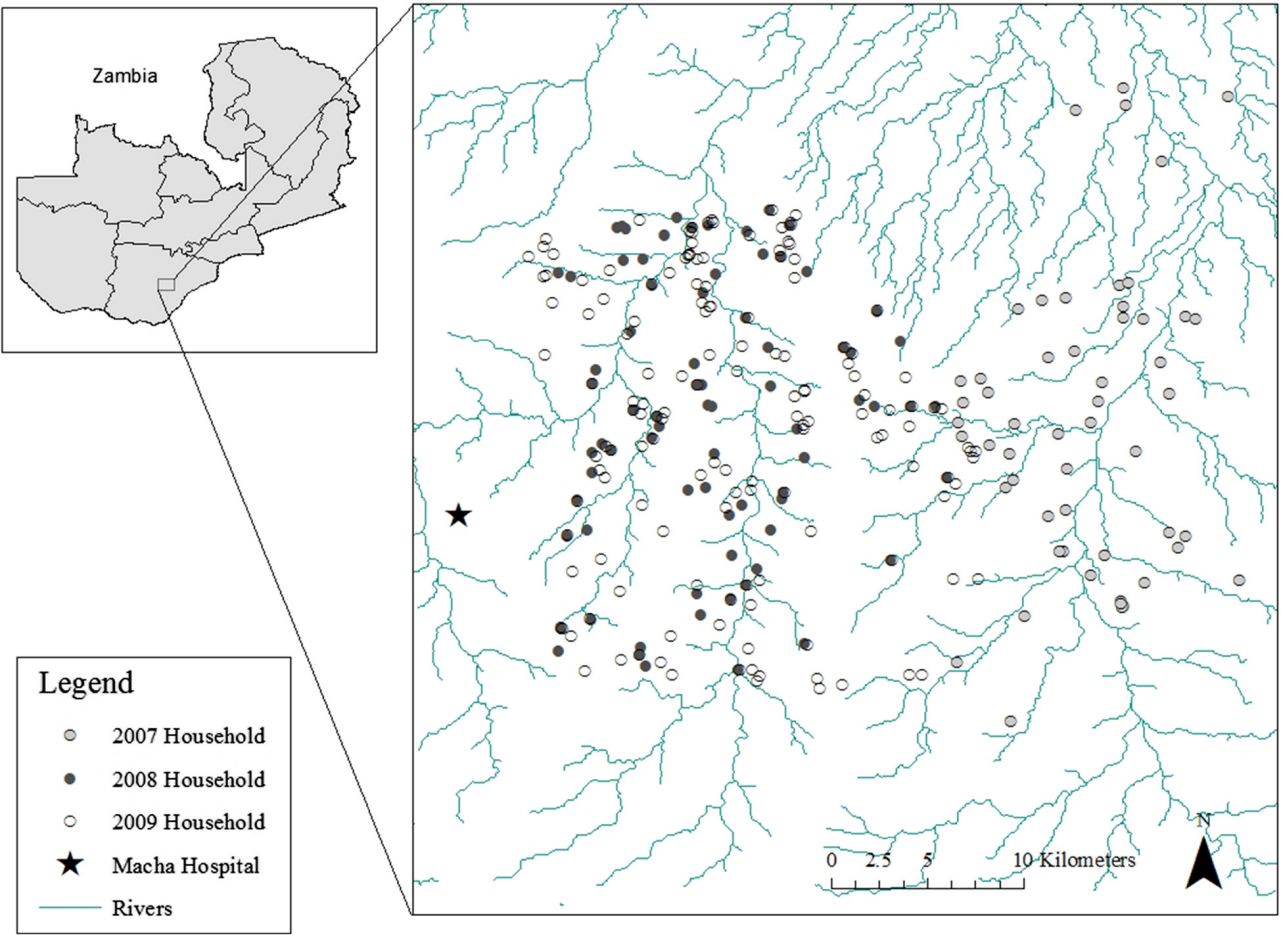
Location of study households in the 2007 and 2008–2009 study areas in Choma District, Southern Province, Zambia.

The study was conducted in two sites within the catchment area of Macha Hospital in Choma District, Southern Province, Zambia between April 2007 and December 2009. Macha Hospital is located approximately 70 km from the nearest town of Choma on a plateau at an altitude of approximately 1,100 meters above sea level and in a habitat characterized as Miombo woodland. A single rainy season, lasting from approximately December through March, is followed by a cool, dry season from April to July, and a hot, dry season from August to November. The catchment area is populated by traditional villagers living in small, scattered homesteads. *Anopheles arabiensis* is the primary vector responsible for malaria transmission [18], which peaks during the rainy season. The study site in 2007 consisted of a 525 km^2^ region to the east of Macha Hospital (Figure 1). In 2008 and 2009, the study site was shifted to a 575 km^2^ area west of the 2007 study site (Figure 1).

The Southern Province of Zambia historically had hyperendemic transmission of *P. falciparum*[19]. More recently, the entomological inoculation rate for *An. arabiensis* was estimated to range from 1.6 to 18.3 infective bites per person per season [18]. Zambia introduced artemether-lumefantrine as first-line anti-malarial therapy in 2002, which reached the Macha Hospital catchment areas in 2004, and insecticide-treated bed nets (ITNs) were widely distributed in Southern Province, Zambia in 2007. Widespread indoor residual spraying has not been conducted in the study areas.

### Study design and methods

Satellite images were used to construct a sampling frame for the random selection of households enrolled in prospective longitudinal and cross-sectional surveys [20]. The sampling frame for the random selection of households was constructed from a Quickbird™ satellite image obtained from DigitalGlobe Services, Inc. (Denver, Colorado). The image was imported into ArcGIS 9.2 (Redlands, CA) and locations of households were identified and enumerated manually. Structures of appropriate size and shape were identified as potential residences. Household locations of cases and controls were collected in the Universal Transverse Mercator, Zone 35 south coordinate system. Selected households were allocated to one of two study cohorts: longitudinal and cross-sectional. Households in the longitudinal cohort were surveyed repeatedly approximately every other month and households in the cross-sectional cohort were surveyed once.

All individuals residing within a selected household were eligible to participate. After obtaining permission from the head of household and individual written informed consent, a questionnaire was administered to each participant older than 16 years of age residing within the household and to parents or guardians of those younger than 16 years of age. Data collected included demographic information, history of recent malaria and anti-malarial treatment, knowledge of malaria transmission and prevention, and the use of ITNs.

A blood sample was collected by finger prick for a rapid diagnostic test (RDT) for malaria and preparation of dried blood spots (DBS). The DBS were collected on filter paper (Whatman, Protein Saver card 903, Piscateway, New Jersey), dried overnight and stored individually with desiccant in a sealed plastic bag at −20°C. DBS collected in 2007 were stored with desiccant at room temperature and DBS collected from February to September in 2008 were initially stored at room temperature but subsequently stored at −20°C. All samples collected after September 2008 were stored at −20°C.

A RDT (ICT Diagnostics, Cape Town, South Africa) was used to detect *P. falciparum* histidine-rich protein 2. This RDT was reported to detect 82% of test samples with *P. falciparum* at a concentration of 200 parasites/μL and 98% of test samples with a concentration of 2000 parasites/μL, with false positives in 0.6% of negative samples [21]. Individuals who were RDT positive were offered treatment with artemether-lumefantrine (Coartem®).

The study was approved by the University of Zambia Research Ethics Committee and the Institutional Review Board at the Johns Hopkins Bloomberg School of Public Health.

### Laboratory methods

Antibodies to whole asexual parasite (NF54) lysate were measured by enzyme immunoassay using serum extracted from DBS. A circle of 7 mm (corresponding to approximately 10 μL blood volume) in diameter was punched out of the DBS and half of the circle (approximately 5 μL blood volume, therefore 2.5 μL of serum) was used to extract serum. The half circle was soaked in 500 μL (1:200 dilution of serum) 5% skim milk in PBS containing 0.05% Tween 20 (PBST) for 1 hour at room temperature. Immulon 2HB flat-bottomed 96-well plates (Thermo, Rochester, New York) were coated with 1 μg/mL of whole *P. falciparum* asexual stage lysate diluted in PBS and incubated overnight at 4°C. Plates were washed with PBST three times, blocked with 5% skim milk in PBS for 1 hour at 37°C, and washed again with PBST four times. Eluted samples were plated in triplicate and incubated for 1 hour at 37°C. Negative control serum (pooled serum from adults who had never been exposed to malaria) and positive control serum (serum from adults who resided in malaria endemic areas of Zimbabwe or Zambia) were diluted 1:200 in 5% skim milk PBST and included in triplicate on each plate. The plates were subsequently washed with PBST four times and incubated for one hour at 37°C with peroxidase-labeled goat anti-human IgG (KPL, Inc., Gaithersburg, Maryland). Following four washes, the plates were incubated for 15 minutes with 100 μL of ABTS solution (KPL). Absorbance was measured at 405 nm and IgG levels were expressed as the optical density.

A threshold optical density (OD) value of 0.57 was established to distinguish seronegative from seropositive individuals based on the mean OD value plus three standard deviations from filter paper spotted with serum from ten individuals never exposed to malaria. Individuals with OD values above this threshold were defined as seropositive. Results were included only if the standard deviation of the triplicate values was within 20% of the mean OD value. The mean standard deviation for the triplicate values was 0.020 in 2008 and 0.026 in 2009.

### Statistical methods for the longitudinal analysis

Associations between RDT positivity and antibody levels to *P. falciparum* antigens, expressed as OD values and adjusted for age, were analyzed using data from participants enrolled in the longitudinal cohort. Malaria infection was defined in two ways: 1) a positive RDT result on the day of the study visit was defined as concurrent malaria; and 2) a positive RDT result at any previous study visit was defined as prior malaria. The change in antibody level over the one year study period was expressed as the mean difference in OD values between study visits. To account for within-subject correlation, a linear regression model was fitted adjusting for within subject correlation, with random intercept and robust standard errors.

### Statistical methods for the spatial analysis

Using the coefficient for the change in the OD value in individuals with a prior documented history of recent malaria, participants enrolled in the longitudinal cohort in 2008 and 2009 were categorized as either a case or control. A case was defined as an individual whose change in OD value from the first to the last study visit at the end of the study year exceeded the coefficient from the linear model, and thus represented someone with a greater than expected increase in OD value and serological evidence of recent exposure to *P. falciparum*. A control was defined as an individual whose change in OD value from the first to the last study visit at the end of the study year did not exceed the coefficient from the linear model. Spatial clustering of cases was analyzed with SatScan™ software (version 9.0) using the Kulldof spatial statistic [22] and a Bernoulli probability model. Coordinates were specified using a Cartesian coordinate system and the maximum spatial cluster size was set at 50% of the population at risk. To detect clusters, SatScan generated circular windows of different sizes. The circular window shape is the most commonly used shape [23,24] and represents the most compact area. The number of cases in each window was compared to the expected number of cases based on the total population size and the total number of cases. The likelihood function was maximized over all scanned windows and the window with the maximum likelihood function was the cluster least likely to arise by chance. The p-value was obtained through Monte Carlo hypothesis testing with 999 simulations.

## Results

### Baseline characteristics of participants

Antibody levels to *P. falciparum* antigens were measured in 234 participants between April and July 2007 (cross-sectional cohort = 90, longitudinal cohort = 144), 435 participants between February and December 2008 (cross-sectional cohort = 317, longitudinal cohort = 118), and 855 participants between January and December 2009 (cross-sectional cohort = 675, longitudinal cohort = 180). Most participants enrolled in the longitudinal cohort in 2008 continued to be followed in 2009, although three longitudinal households were replaced and six new households were added in 2009. The median age of the study participants was 12.2 years (interquartile range [IQR]: 5.08, 29.9) in 2007, 14.2 years (IQR: 6.34, 34.2) in 2008, and 14.0 years (IQR: 6.34, 32.1) in 2009). Slightly less than half of the participants were male (47%) (Table 1). The prevalence of RDT positivity differed markedly by study location and year: 24% of the baseline population (cross-sectional cohort plus the first visit of longitudinal cohort) was RDT positive in the eastern area in 2007, compared to 8.1% and 1.4% in the western area in 2008 and 2009, respectively.

**Table 1:** Characteristics of the study populations in 2007 and 2008-2009

	**2007**		**2008**		**2009**	
	**Cross-Sectional**	**Longitudinal (1**^**st **^**visit)**	**Cross-Sectional**	**Longitudinal (1**^**st **^**visit)**	**Cross- Sectional**	**Longitudinal (1**^**st **^**visit)**
Number of participants	90	144	317	118	675	180
Median age in years (range)	12.8 (0.50-83)	12.1 (0.21-86)	14.2 (0.25-84)	14.3 (0.39-78)	14.0 (0.07-85)	13.0 (0.23-79)
Male (%)	44 (49)	63 (44)	149 (47)	58 (49)	322 (48)	85 (47)
RDT positive (%)	29 (32.2)	37 (25.7)	31 (9.78)	4 (3.39)	10 (1.48)	2 (1.11)

### Seropositivity increased with age

**Figure 2:**
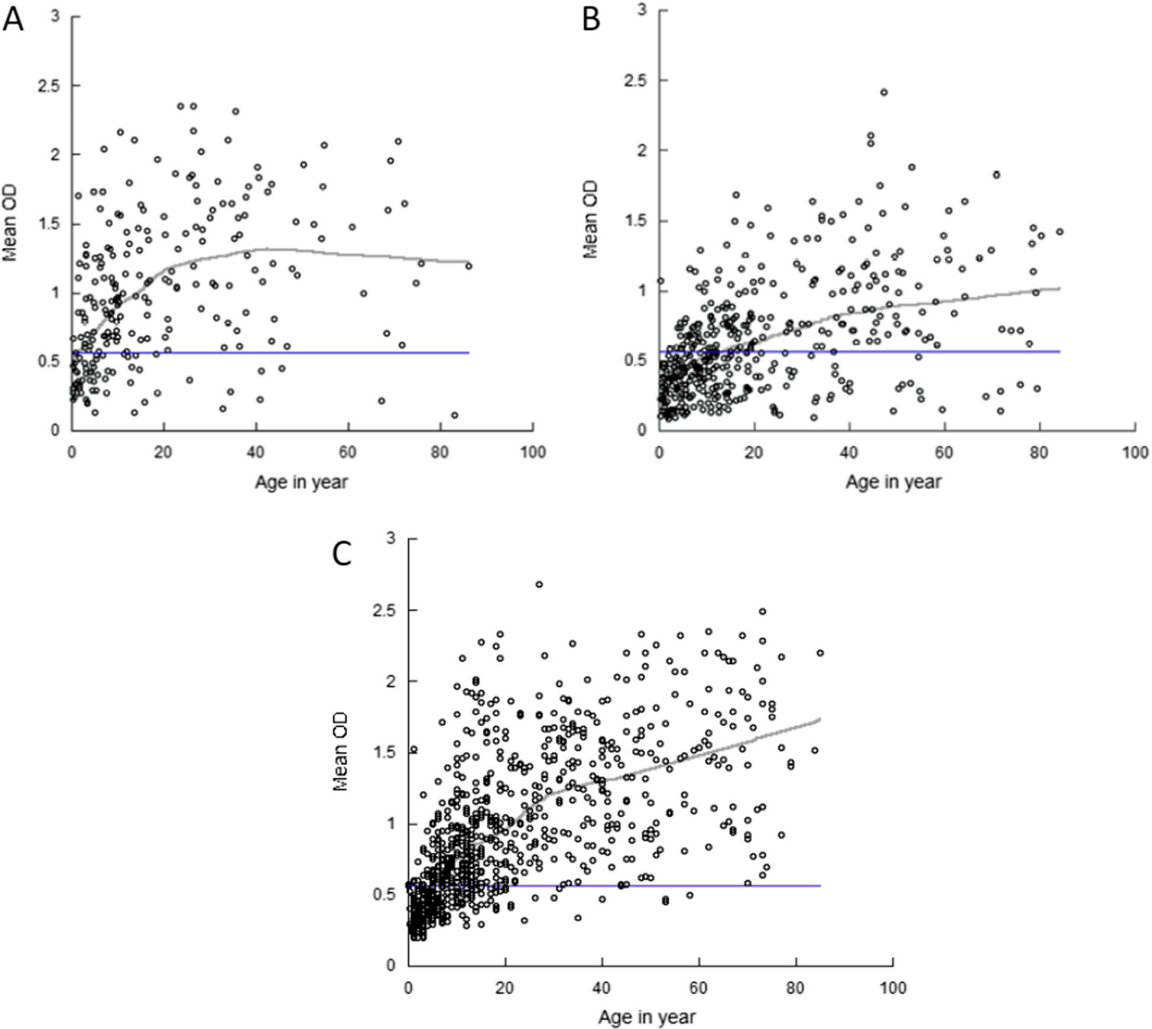
**IgG antibody levels to *P. falciparum *whole antigen expressed as optical density values by age: A. 2007, B. 2008 and C. 2009. **A Lowess curve was fit to the data. The straight line indicates the threshold OD value established using the mean OD value plus three standard deviations.

IgG antibody levels to *P. falciparum* were measured in the baseline study population consisting of participants in the cross-sectional cohort and the first visit of the longitudinal cohort. In each year, the proportion of seropositive individuals increased with age (Figure 2). Consistent with the differences in parasite prevalence, the proportion of seropositive children younger than five years of age was lower in the 2008 and 2009 study areas than in the 2007 study area (34% in 2007, 11% in 2008 and 12% in 2009).

### RDT positivity was positively correlated with higher serologic responses

The effect of documented malaria during the study period on antibody levels to *P. falciparum* antigens was assessed among participants in the longitudinal cohort. In 2007, 114 individuals enrolled in the longitudinal cohort had at least two study visits. Of these, 47 had at least one RDT positive result, including 15 participants with two RDT positive results and two participants with three RDT positive results. To test the effect of having multiple RDT positive results during prior visits, the OD values at the end of 2007 study year among participants with a single positive RDT (mean OD = 1.29) was compared to participants with two or three RDT positive results (mean OD = 1.09; *p* = 0.66). As there was no statistically significant difference in mean EIA OD between individuals with a single or multiple RDT positive results, RDT results during any prior study visits were dichotomized as negative (no prior positive RDT result) or positive (at least one prior RDT positive result) for the analysis.

In 2008, 99 individuals enrolled in the longitudinal cohort had at least two study visits. Of these, nine participants had a single RDT positive result and one participant had two RDT positive results. In 2009, 168 individuals enrolled in the longitudinal cohort had at least two study visits. Only two were RDT positive and none had multiple RDT positive results. Due to the small number of RDT positive results in 2009, the association between RDT positivity and antibody levels to *P. falciparum* antigens was not examined.

The association between the antibody levels to *P. falciparum* antigens and a concurrent or prior documented episode of malaria was assessed, adjusting for age. In 2007, participants with a positive RDT result at the concurrent visit had a 0.177 higher OD value (95% CI: 0.063, 0.292, p = 0.002) than RDT negative participants, adjusting for age (Table 2). In 2008, participants with a positive RDT result at the concurrent visit had a statistically non-significant 0.063 lower OD value (95% CI: -0.245, 0.119, p = 0.51) than RDT negative participants after adjusting for age (Table 2). Participants with at least one positive RDT result at any prior visit during the study period had a 0.261 higher OD value (95% CI: 0.090, 0.432, p = 0.003) in 2007 and a 0.116 higher OD value (95% CI: 0.009, 0.222, p = 0.03) in 2008 compared to RDT negative participants after adjusting for age (Table 2).

**Table 2:** Association between RDT positivity at the concurrent and prior visit and EIA OD values adjusting for age, 2007 and 2008

	**2007**		**2008**	
	**Coefficient**	**p-value**	**Coefficient**	**p-value**
Positive RDT result at the concurrent study visit	0.177	0.002	-0.063	0.50
Positive RDT result at the prior study visit	0.261	0.003	0.116	0.03

### Spatial clustering of households comprised of individuals with serologic evidence of recent malaria

The coefficient for the OD value from the linear model for individuals having at least one positive RDT result at any prior visit during the study period in 2008 (β = 0.116), was used to assess whether serology could be used to identify spatial clusters of malaria transmission in the study area for 2008 and 2009. In 2008, 55 participants had samples collected at the beginning (February) and end (December) of the study period. The median age at the first study visit was 11 years (IQR: 6, 33) and the median interval between the first and the last study visit was 296 days (range: 284 to 305 days). Of these 55 participants, 43 (78%) with a median age of 14 years (IQR: 7, 36) had OD values that increased more than 0.116. In 2009, 74 individuals had samples collected at the beginning (January) and end (December) of the study period. The median age at the first study visit was 13 years (IQR: 5.6, 35) and the median interval between the first and last study visit was 305 days (range: 281 to 324 days). Of these 74 participants, 10 (14%) with a median age of 21 years (IQR: 7.4, 30) had an OD values that exceeded 0.116.

**Figure 3:**
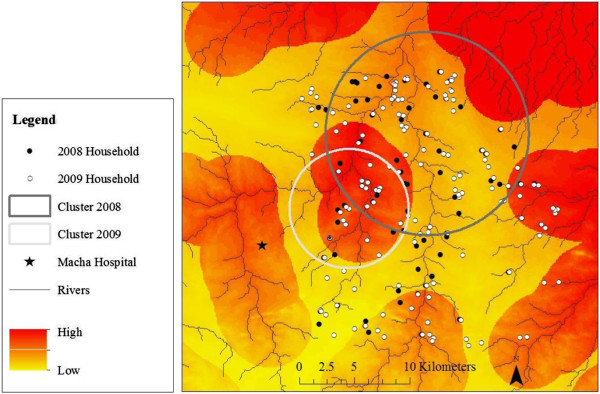
**Spatial clusters of recent malaria cases identified serologically and ecological predictors of malaria transmission. **Study households are shown in black (2008) and white (2009) dots. Spatial clusters of households comprising residents with serologic evidence of recent malaria infection are shown in the large circles (2008 = grey; 2009 = light grey). The background consists of a malaria risk map based on ecological features of the terrain [20], with the darker red indicating a higher risk of malaria.

Participants with OD values that exceeded 0.116 over the one year study period were classified as cases and clusters of households with cases were identified. The primary household clusters identified in 2008 (within a 9.2 km radius) were not statistically significant (p = 0.15), but household clusters identified in 2009 (within a 5.4 km radius) achieved marginal statistical significance (p = 0.06) (Table 3). Study households were overlaid on a malaria risk map generated from ecological characteristics of the terrain [20] to assess whether spatial clusters of recent malaria cases identified serologically were consistent with ecological predictors of malaria transmission in the study area (Figure 3). The cluster of households identified serologically in 2009 overlapped a region previously identified to be at risk for malaria transmission (Figure 3).

**Table 3:** Spatial clusters of households with positive cases more than expected, 2008 and 2009

**Year**	**Total population in the cluster**	**Observed number of positive cases**	**Expected number of positive cases**	**Relative risk**	**p-value**
**2008**	27	25	21.11	1.44	0.15
**2009**	34	9	4.59	10.59	0.06

## Discussion

Antibody levels to whole, asexual stage *P. falciparum* antigens measured by enzyme immunoassay increased with increasing age and were correlated with prior malaria infection as documented by RDT in this region of declining malaria transmission in southern Zambia. In the year with the lowest parasite prevalence, spatial clustering of individuals with serologic evidence of recent infection overlapped with a region at risk for malaria transmission as previously determined by ecological factors [20]. These findings suggest that serology may be a useful tool for monitoring malaria transmission in regions of declining malaria burden with low levels of parasite prevalence.

Residents of malaria endemic areas acquire antibodies to *P. falciparum* and clinical immunity to malaria after repeated exposure, with earlier acquisition of seropositivity and clinical immunity in regions with higher transmission intensity [25]. Immunity to clinical malaria and seropositivity increase with increasing age due to cumulative exposure to malaria parasites; however, protective immunity may wane in the absence of repeated exposure to malaria parasites [26]. In southern Zambia, antibody levels demonstrated age-dependent increases consistent with other studies [10,11]. Although antibodies to whole parasite lysate were measured, prior reports demonstrated that antibody responses to whole schizont extract were comparable to antibody responses to single antigens such as AMA1 and MSP-1_19_[14].

The use of serology as a marker of malaria transmission intensity and recent exposure to *P. falciparum* was recently described [10]. A potential disadvantage of serology to track changes in malaria transmission is that antibody responses to *P. falciparum* may not be suitable to detect short term changes in population immunity in regions with rapid declines in malaria transmission. This study attempted to identify recent exposure to *P. falciparum* by measuring an increase in OD values above the expected value for having RDT positive result during one year study period. The findings suggest that recent exposure to *P. falciparum* can be detected through increases in OD values. Prior studies measured antibody responses to single parasite antigens, such as AMA1 or MSP1_19_, and samples were collected in cross-sectional studies [9,11-13]. In contrast, we were able to measure serological responses in a longitudinal cohort and adjusted for within-individual heterogeneity.

Spatial clusters of individuals with serological evidence of recent infection was identified and these clusters were validated by comparing them to a previously reported malaria risk map of the study area [20]. Prior studies identified clusters of malaria transmission using serology but the reported clusters were smaller (< 1 km) than the clusters identified in southern Zambia (> 5 km) [27,28]. The small sizes of the clusters were attributed to the estimated distance traversed by the mosquito vector [29]. In these studies, antibodies to specific antigens such as MSP1_19_, AMA1 and MSP2 were measured; the broader antibody response to whole parasite lysate in our study may account for the larger cluster sizes.

## Conclusions

Antibodies to whole, asexual stage *P. falciparum* antigens were positively correlated with prior RDT positivity. Serological surveys to whole asexual *P. falciparum* antigens using blood collected as dried blood spots can be used to detect recent malaria infection and identify focal areas of transmission, particularly in a region of declining malaria burden.

## Competing interests

The authors declare that they have no competing interests.

## Authors’ contributions

TK participated in the coordination of the study, performed the data analysis and drafted the manuscript. SC performed the enzyme immunoassays and reviewed the manuscript. TS participated in the spatial analyses and reviewed the manuscript. HH coordinated the data and specimen collection and reviewed the manuscript. SM supervised the laboratory assays and reviewed the manuscript. PET supervised the field activities and reviewed the manuscript. GG participated in the spatial analyses and reviewed the manuscript. WJM conceived of the study, participated in its design and coordination, and participated in the preparation of the manuscript. All authors read and approved the final manuscript.

## References

[B1] Barnes KI Chanda P Ab Barnabas G Impact of the large-scale deployment of artemether/lumefantrine on the malaria disease burden in Africa: case studies of South Africa, Zambia and Ethiopia Malar J 2009 8 Suppl 1 S8 10.1186/1475-2875-8-S1-S8 19818175 PMC2760243

[B2] O'Meara WP Mangeni JN Steketee R Greenwood B Changes in the burden of malaria in sub-Saharan Africa Lancet Infect Dis 2010 10 545 555 10.1016/S1473-3099(10)70096-7 20637696

[B3] Chizema-Kawesha E Miller JM Steketee RW Mukonka VM Mukuka C Mohamed AD Miti SK Campbell CC Scaling up malaria control in Zambia: progress and impact 2005–2008 Am J Trop Med Hyg 2010 83 480 488 10.4269/ajtmh.2010.10-0035 20810807 PMC2929038

[B4] Government of the Republic of Zambia, Ministry of Health Zambia national malaria indicator survey 2010 2011 http://www.nmcc.org.zm/files/FullReportZambiaMIS2010_001.pdf

[B5] Steketee RW Sipilanyambe N Chimumbwa J Banda JJ Mohamed A Miller J Basu S Miti SK Campbell CC National malaria control and scaling up for impact: the Zambia experience through 2006 Am J Trop Med Hyg 2008 79 45 52 18606763

[B6] World Health Organization Malaria deaths decline by 66% in Zambia 2009 Geneva: WHO

[B7] World Health Organization World malaria report 2010 2011 Geneva: WHO

[B8] Corran P Coleman P Riley E Drakeley C Serology: a robust indicator of malaria transmission intensity? Trends Parasitol 2007 23 575 582 10.1016/j.pt.2007.08.023 17988945

[B9] Drakeley CJ Corran PH Coleman PG Tongren JE McDonald SL Carneiro I Malima R Lusingu J Manjurano A Nkya WM Lemnge MM Cox J Reyburn H Riley EM Estimating medium- and long-term trends in malaria transmission by using serological markers of malaria exposure Proc Natl Acad Sci USA 2005 102 5108 5113 10.1073/pnas.0408725102 15792998 PMC555970

[B10] Drakeley C Cook J Potential contribution of sero-epidemiological analysis for monitoring malaria control and elimination: historical and current perspectives Adv Parasitol 2009 69 299 352 19622411 10.1016/S0065-308X(09)69005-9

[B11] Stewart L Gosling R Griffin J Gesase S Campo J Hashim R Masika P Mosha J Bousema T Shekalaghe S Cook J Corran P Ghani A Riley EM Drakeley C Rapid assessment of malaria transmission using age-specific sero-conversion rates PLoS One 2009 4 e6083 10.1371/journal.pone.0006083 19562032 PMC2698122

[B12] Cook J Reid H Iavro J Kuwahata M Taleo G Clements A McCarthy J Vallely A Drakeley C Using serological measures to monitor changes in malaria transmission in Vanuatu Malar J 2010 9 169 10.1186/1475-2875-9-169 20553604 PMC2904786

[B13] Cook J Kleinschmidt I Schwabe C Nseng G Bousema T Corran PH Riley EM Drakeley CJ Serological markers suggest heterogeneity of effectiveness of malaria control interventions on Bioko Island, equatorial Guinea PLoS One 2011 6 e25137 10.1371/journal.pone.0025137 21980386 PMC3181341

[B14] Akpogheneta OJ Duah NO Tetteh KK Dunyo S Lanar DE Pinder M Conway DJ Duration of naturally acquired antibody responses to blood-stage Plasmodium falciparum is age dependent and antigen specific Infect Immun 2008 76 1748 1755 10.1128/IAI.01333-07 18212081 PMC2292892

[B15] Corran PH Cook J Lynch C Leendertse H Manjurano A Griffin J Cox J Abeku T Bousema T Ghani AC Drakeley C Riley E Dried blood spots as a source of anti-malarial antibodies for epidemiological studies Malar J 2008 7 195 10.1186/1475-2875-7-195 18826573 PMC2567984

[B16] Estevez PT Satoguina J Nwakanma DC West S Conway DJ Drakeley CJ Human saliva as a source of anti-malarial antibodies to examine population exposure to Plasmodium falciparum Malar J 2011 10 104 10.1186/1475-2875-10-104 21527045 PMC3112448

[B17] Chidi AP Chishimba S Kobayashi T Hamapumbu H Mharakurwa S Thuma PE Moss WJ Validation of oral fluid samples to monitor serological changes to Plasmodium falciparum: an observational study in southern Zambia Malar J 2011 10 162 10.1186/1475-2875-10-162 21663660 PMC3141589

[B18] Kent RJ Thuma PE Mharakurwa S Norris DE Seasonality, blood feeding behavior, and transmission of Plasmodium falciparum by Anopheles arabiensis after an extended drought in southern Zambia Am J Trop Med Hyg 2007 76 267 274 17297034 PMC4152308

[B19] Mharakurwa S Thuma PE Norris DE Mulenga M Chalwe V Chipeta J Munyati S Mutambu S Mason PR Malaria epidemiology and control in Southern Africa Acta Trop 2012 121 202 206 10.1016/j.actatropica.2011.06.012 21756864 PMC3214248

[B20] Moss WJ Hamapumbu H Kobayashi T Shields T Kamanga A Clennon J Mharakurwa S Thuma PE Glass G Use of remote sensing to identify spatial risk factors for malaria in a region of declining transmission: a cross-sectional and longitudinal community survey Malar J 2011 10 163 10.1186/1475-2875-10-163 21663661 PMC3123248

[B21] World Health Organization Malaria rapid diagnostic test performance. Results of WHO product testing of malaria RDTs: round 1 2008 http://www.who.int/tdr/publications/tdr-research-publications/rdt3_summary.pdf

[B22] Kulldorff M Nagarwalla N Spatial disease clusters: detection and inference Stat Med 1995 14 799 810 10.1002/sim.4780140809 7644860

[B23] Haque U Hashizume M Sunahara T Hossain S Ahmed SM Haque R Yamamoto T Glass GE Progress and challenges to control malaria in a remote area of Chittagong hill tracts, Bangladesh Malar J 2010 9 156 10.1186/1475-2875-9-156 20537127 PMC2910016

[B24] Loha E Lunde TM Lindtjorn B Effect of bednets and indoor residual spraying on spatio-temporal clustering of malaria in a village in South ethiopia: a longitudinal study PLoS One 2012 7 e47354 10.1371/journal.pone.0047354 23077598 PMC3470588

[B25] Doolan DL Dobano C Baird JK Acquired immunity to malaria Clin Microbiol Rev 2009 22 13 36 Table of Contents 10.1128/CMR.00025-08 19136431 PMC2620631

[B26] Ghani AC Sutherland CJ Riley EM Drakeley CJ Griffin JT Gosling RD Filipe JA Loss of population levels of immunity to malaria as a result of exposure-reducing interventions: consequences for interpretation of disease trends PLoS One 2009 4 e4383 10.1371/journal.pone.0004383 19198649 PMC2634959

[B27] Bousema T Drakeley C Gesase S Hashim R Magesa S Mosha F Otieno S Carneiro I Cox J Msuya E Kleinschmidt I Maxwell C Greenwood B Riley E Sauerwein R Chandramohan D Gosling R Identification of hot spots of malaria transmission for targeted malaria control J Infect Dis 2010 201 1764 1774 10.1086/652456 20415536

[B28] Bejon P Williams TN Liljander A Noor AM Wambua J Ogada E Olotu A Osier FH Hay SI Farnert A Marsh K Stable and unstable malaria hotspots in longitudinal cohort studies in Kenya PLoS Med 2010 7 e1000304 10.1371/journal.pmed.1000304 20625549 PMC2897769

[B29] Bousema T Griffin JT Sauerwein RW Smith DL Churcher TS Takken W Ghani A Drakeley C Gosling R Hitting hotspots: spatial targeting of malaria for control and elimination PLoS Med 2012 9 e1001165 10.1371/journal.pmed.1001165 22303287 PMC3269430

